# Oscillometry Assesses Small Airway Disease and Reveals Peripheral Lung Pathology in Early Pulmonary Fibrosis: A Cross-Sectional Study

**DOI:** 10.3390/diagnostics14242873

**Published:** 2024-12-20

**Authors:** Athena Gogali, Georgia Gkrepi, Christos Kyriakopoulos, Konstantinos Tatsis, Konstantinos Katsoulis, Chara Tselepi, Konstantinos Kostikas

**Affiliations:** 1 Respiratory Medicine Department, University of Ioannina, 45110 Ioannina, Greece; geogkrepi@gmail.com (G.G.); ckyriako@yahoo.gr (C.K.); konstantatsis@gmail.com (K.T.); haratselepi@gmail.com (C.T.); ktkostikas@gmail.com (K.K.); 2 Respiratory Medicine Department, 424 Army General Hospital, 56429 Thessaloniki, Greece; kostascmax@gmail.com

**Keywords:** interstitial lung disease, small airway dysfunction, oscillometry, R5-19

## Abstract

**Background/Objectives:** Small airway disease/dysfunction (SAD) is crucial in obstructive airway diseases but is less investigated in interstitial lung disease (ILD). There are only a few physiological studies investigating SAD in the context of pulmonary fibrosis. Oscillometry is a simple technique that assesses SAD with minimal patient effort. In this study, we investigated the role of oscillometry in patients with mild pulmonary fibrosis without evident obstructive disorder, focusing on small airways. **Methods:** Oscillometry and pulmonary function test (PFT) data of consecutive patients newly diagnosed with pulmonary fibrosis of unknown etiology in a university hospital ILD clinic were collected and analyzed. **Results:** Data from 34 patients with mild pulmonary fibrosis were collected in 6 months. Disease severity, as evaluated by FVC, presented strong correlations with the oscillometry parameters: resistance (R5: r = −0.588, *p* < 0.001), reactance (X5: r = 0.671, *p* < 0.001), resonant frequency (Fres: r = −0.562, *p* = 0.001), and the area of reactance (AX: r = −0.515, *p* = 0.002). The oscillometry parameter R5-19-expressing was abnormal in 27% of patients, correlated with FEF25-75% (r = −0.370, *p* = 0.021) and was a predictor of a FEF25-75% < 60% pred. with AUC 0.738 (95%Cl 0.519–0.956). R5-19 correlated with FVC (r = −0.481, *p* = 0.004) and was the only SAD parameter that correlated with the composite physiologic index (CPI, r = 0.338, *p* = 0.04), while FEF 25-75% and RV/TLC% did not. **Conclusions:** Oscillometry is an easy to perform technique that may reveal early mechanical alterations caused by pulmonary fibrosis. Peripheral resistance, as expressed by R5-19, which identifies small airway dysfunction as a marker of peripheral lung pathology, may be complementary to pulmonary function testing and may also have prognostic implications for ILD patients.

## 1. Introduction

Small airways, airways with a diameter of 2 mm or less, are often called the “silent zone” of the lung, mainly because they are affected early in the course of disease before any symptoms or spirometric changes occur [1]. Although small airway disease/dysfunction (SAD) is a fundamental element of chronic obstructive pulmonary disease (COPD) and asthma, this factor is less investigated in interstitial lung disease (ILD), where findings are mainly focused on interstitial and alveolar abnormalities [2]. Recent studies combining pathology and HRCT observations have demonstrated that the perception that idiopathic pulmonary fibrosis (IPF) spares the airways is not quite right, as regions of minimal fibrosis are associated with a significant reduction in the number of terminal bronchioles, while the remainder exhibit wall thickening and lumen distortion [3]. There are only a few physiological studies that have investigated SAD in the context of pulmonary fibrosis. A small number of previous studies have shown that spirometry-defined SAD is a feature of advanced fibrosis and correlates with decreased survival, especially when it is combined with obstructive disease [4,5].

Oscillometry is a simple technique that assesses small airway dysfunction (SAD) with minimal patient effort [6]. Forced oscillation technique (FOT) assesses the mechanical properties of the lung and evaluates pulmonary obstruction by measuring the respiratory system’s response to small-pressure stimuli generated by a loudspeaker during normal breathing [7]. Oscillometry provides additional valuable information about lung periphery, the first area affected in pulmonary fibrosis, including the compliance of the lung parenchyma and the small airways.

Therefore, in this pilot study, we investigated whether oscillometry provides complementary data to pulmonary function tests (PFTs) in patients with mild pulmonary fibrosis without evident obstructive disorder, focusing on small airways.

## 2. Materials and Methods

In this prospective observational pilot study, we evaluated consecutive patients with newly diagnosed idiopathic fibrosing ILD in our tertiary ILD clinic, which is a referral university hospital clinic, in a 6-month period. All participants had a predominantly fibrotic pattern on high-resolution computerized tomography (HRCT) of less than 20% extent. HRCT scans were scored according to the easily applicable limited/extensive staging system reported by Goh et al. [8]. Two experienced clinicians (A.G. and K.K.) meticulously evaluated the study population and excluded individuals with ILDs of known etiology (e.g., in the context of collagen vascular disease or hypersensitivity pneumonitis) and also patients with asthma, COPD, or radiological signs of airway obstruction such as mosaic attenuation, air trapping, emphysema, and centrilobular nodules. This study was performed in accordance with the recommendations of the Declaration of Helsinki, the International Conference of Harmonisation—Good Clinical Practice (ICH-GCP) Guidelines, the EU-Directive 2001/20, and all national requirements and was approved by the Scientific Council of our hospital. Written informed consent was obtained from all participants prior to their inclusion in this study.

All patients underwent FOT (RESMON pro Full-V3, MCG Diagnostics, Saint Paul, MN, USA) and full pulmonary function tests (PFT, Vyntus Body Plethysmograph, Vyaire Inc., Mettawa, IL, USA). The forced expiratory flow rate between 25 and 75% of forced vital capacity (FVC) (FEF25-75%) and the ratio of residual volume (RV) to total lung capacity (TLC) (RV/TLC) were assessed as SAD indices. The oscillometry parameters resistance (R5), reactance (X5), resonant frequency (Fres), and the area of reactance (AX) were evaluated, while resistance heterogeneity measured between 5 and 19 Hz (R5-19) assessed SAD. Specifically, R5-19 was considered the primary oscillometry parameter indicative of SAD. We also calculated the composite physiologic index (CPI), an index that reflects the extent of pulmonary fibrosis better than individual pulmonary function tests and is associated with prognosis including mortality [9].

Descriptive statistics were presented as mean (SD), and correlations were performed with Spearman’s rank correlation coefficient. Correlations between FOT and PFT were investigated. Statistical analysis was performed with SPSS (Version 22; IBM, Chicago, IL, USA) and GraphPad Prism v7 software (GraphPad, Inc., San Diego, CA, USA). *p*-values < 0.05 were considered statistically significant.

## 3. Results

Data from a total of 34 patients newly diagnosed with lung fibrosis of unknown etiology were collected in the six months of the study; 19 (56%) were men, with a mean (standard deviation, SD) age of 71.4 (6.7) years and a mean BMI of 28.8 (4.8) kg/m^2^, while half of them (*n* = 17) were current or ex-smokers with a mean history of 19.1 (28.2) pack-years. The majority received an IPF diagnosis (29 patients) according to the Official ATS/ERS/JRS/ALAT Clinical Practice Guideline [10], 3 had nonspecific interstitial pneumonia (NSIP, this pattern was proven histologically), and 2 were characterized as having unclassifiable lung fibrosis.

Overall, our patients exhibited mild ILD at diagnosis with an FVC 89.8 (20.7)% pred. and diffusing capacity (DLCO) 66.5 (23.6)% pred. Additional spirometric parameters included forced expiratory volume in 1 s (FEV1), 2.20 (0.62) L [92.7 (21.2)% pred.]; FEV1/FVC, 81.6 (5.4)%; DLCO/VA, 93.9 (26.7)%; and TLC, 73.0 (14.5)% pred. The SAD values were FEF25-75%, 85.6 (30.1)% pred.; FEF25-75%, 2.31 (0.73) L; RV/TLC%, 88.1 (14.8)% pred.; and RV/TLC, 38.0 (5.5)%. The oscillometry measurements were R5 act., 3.00 (1.10) cmH_2_O/L/s; R5, 93.12 (37.78)% pred.; X5 act., −1.84 (0.95) cmH2O/L/s; X5, 139.28 (84.8)% pred.; Fres act, 14.97 (5.13) Hz; Fres, 117.4 (53.7)% pred.; AX act., 9.22 (9.03) cmH_2_O/L; AX, 230.6 (211.9)% pred.; R19 act., 4.98 (13.59) cmH_2_O/L/s; R19, 85.7 (21.3)% pred.; R5-19, act. 0.38 (0.28) cmH_2_O/L/s; and R5-19, 94.9 (84.2)% pred. A total of 27% of the patients had abnormal values of R5-19, using a threshold of 0.7 cmH_2_O/L/s, as proposed previously [11].

**Figure 1:**
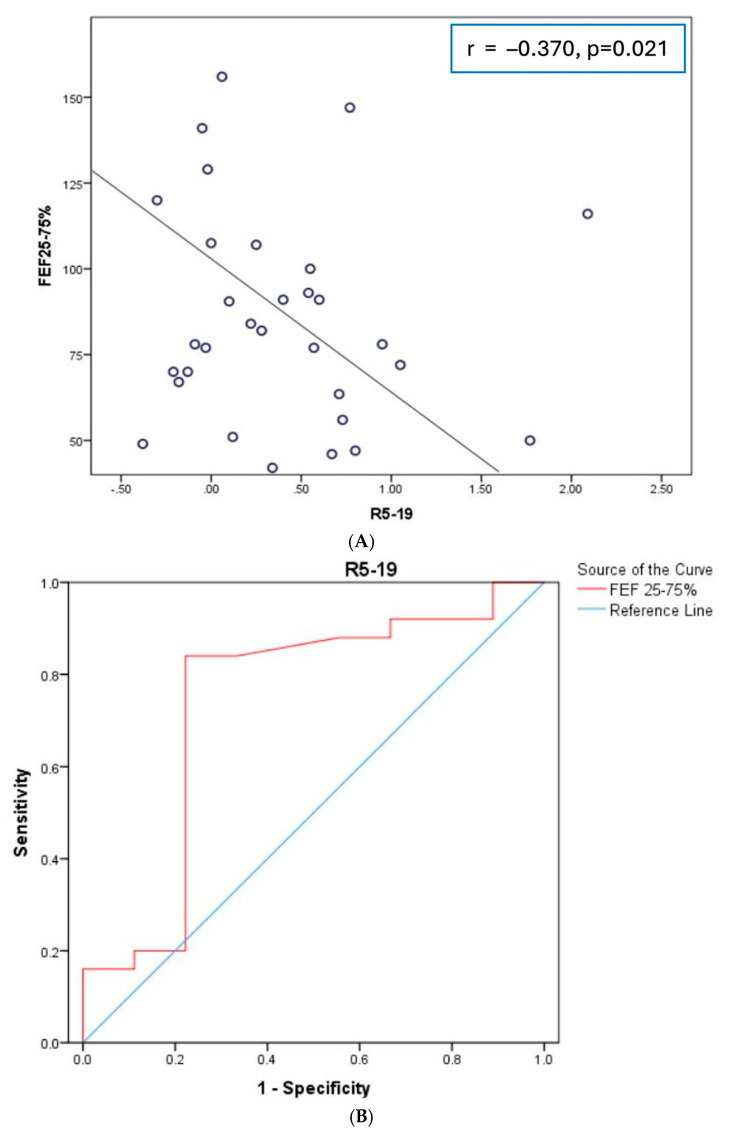

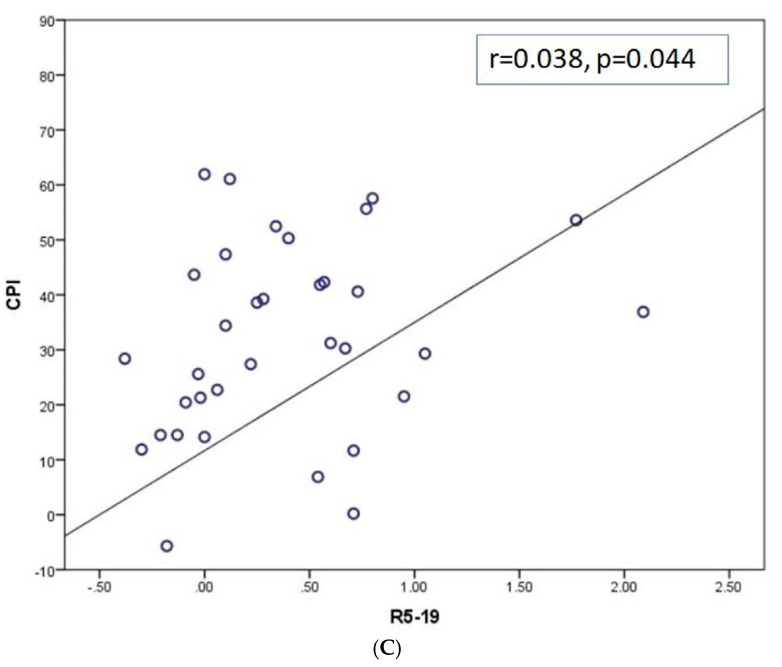
(**A**) Correlation of R5-19 with FEF25-75%; (**B**) receiver operating characteristic (ROC) analysis evaluating the performance of R5-19 in the identification of small airway disease as expressed by FEF25-75% < 60% pred.; (**C**) correlation of R5-19 with the composite physiologic index (CPI).

The correlations between oscillometry parameters and PFT parameters are shown in Table 1. Disease severity, as evaluated by FVC, presented strong correlations with the oscillometry parameters: resistance (R5: r = −0.588, *p* < 0.001), reactance (X5: r = 0.671, *p* < 0.001), resonant frequency (Fres: r = −0.562, *p* = 0.001), and the area of reactance (AX: r = −0.515, *p* = 0.002). The oscillometry parameters also correlated with FEV1 but not FEV1/FVC%. The SAD FOT parameter R5-19 was negatively correlated with FEF25-75% (r = −0.370, *p* = 0.021; Figure 1A) and had an acceptable performance in the prediction of small airway disease, with FEF25-75% < 60% as the “gold standard” in a ROC analysis (AUC 0.738, 95%Cl 0.519–0.956) (Figure 1B). R5-19 did not correlate with smoking history but correlated with disease severity assessed by FVC (r = −0.481, *p* = 0.004). Furthermore, R5-19 was the only SAD parameter that correlated with the composite physiologic index (CPI, r = 0.338, *p* = 0.04) (Figure 1C), while FEF25-75% (r = −0.089, *p* = 0.619) and RV/TLC% (r = −0.111, *p* = 0.539) did not.

## 4. Discussion

In the present study, the oscillometric parameters correlated with ILD disease severity as assessed by FVC, and R5-19 had a good performance in the prediction of spirometric SAD as expressed by FEF25-75%. More importantly, R5-19 was the only SAD parameter related to CPI, a marker of disease prognosis. This study provides evidence for the potential use of oscillometry in the evaluation of disease severity and lung periphery in interstitial lung disease.

Although small airway disease is a major characteristic of COPD and this feature is less investigated in ILD, recent studies based on combined pathology and HRCT observations have demonstrated that airways are often not spared in pulmonary fibrosis. Small airway disease seems to represent an early pathologic feature of idiopathic pulmonary fibrosis, as regions of minimal fibrosis are associated with a significant reduction in the number of terminal bronchioles, while the remaining exhibit major alterations [12]. Furthermore, abnormal small airways exhibited increased expression of matrix metalloproteinases 7 and 9 in the bronchial epithelium [13]. These findings highlight the potential pathogenetic role in lung remodeling of small airways in ILD and raise treatment implications.

SAD may not be easy to assess, and many methods have been developed, including spirometry, body plethysmography, inert gas washout techniques, and imaging modalities with various concerns in terms of availability, complexity, and reproducibility [14]. In our study, oscillometry was evaluated as the main modality to assess SAD in patients with early pulmonary fibrosis. Oscillometry is a novel, easy technique evaluating proximal and distal airway function without performing a forced expiratory maneuver. This has a particular utility in children, the elderly, and patients with severe respiratory disease or specific contraindications, as it requires minimal effort [15]. Oscillometry was proven to be more sensitive than spirometry in detecting SAD in populations exposed to the World Trade Center collapse on 11 September 2001; spirometry, including measures of midexpiratory airflow rates, remained within normal limits, while small airway abnormality was correlated with severity and frequency of wheeze and was independently associated with the presence of systemic inflammation confirmed on histologic evaluation [16]. The value of oscillometry has been more extensively investigated in obstructive diseases. Previous studies have shown that oscillometry may be a more sensitive marker of SAD than FEF25-75% in asthma and COPD with preserved pulmonary function, as patients with oscillometry-defined SAD had a significantly higher incidence of respiratory symptoms compared to those with SAD defined by spirometry [17]. Furthermore, oscillometry data contribute to more accurate characterization of asthma phenotype [18].

Our results indicate that oscillometric parameters correlate with ILD severity, as assessed by FVC, even in mild ILD, consistent with previous reports in more advanced fibrotic disease. Using the method described by Horita and colleagues [19] to identify potential differences in the r values in different correlations between oscillometric and pulmonary function testing parameters, we were not able to identify differences that exceeded the minimal clinically important difference. In a cohort of eighty Japanese patients with IPF that underwent high-resolution computed tomography (HRCT) and aimed to assess the utility of oscillometry as a potential marker for traction bronchiectasis and airflow obstruction, spirometry and oscillometry were investigated in relation to fibrosis-related HRCT findings [20]. FVC correlated negatively with oscillometric parameters and HRCT scores, while respiratory reactance correlated positively with all fibrosis-related HRCT scores [19]. Zhiang et al. showed that spirometry-defined SAD was associated with significantly increased risk of mortality in patients with IPF (HR 1.73, 95% CI 1.02–2.92) [5]. In a retrospective analysis, Yin and colleagues demonstrated that one-third of IPF patients had SAD, assessed by spirometric indices; these patients had a significantly shorter survival compared to non-SAD patients. Histopathological examination in those undergoing lung transplantation presented various degrees of airway distortion and obliteration in triple the number of patients than were captured by spirometry [4]. Finally, Mikamo et al. found that oscillometry was more sensitive than spirometry in the detection of HRCT-defined SAD [21]. Ninety patients with different ILDs were evaluated according to the HRCT findings (mosaic attenuation, air trapping, and centrilobular micronodules) as having or not having SAD. Although pulmonary function parameters (%FVC, %FEV1, FEV1/FVC, FEF 25-75, IC, TLC, FRC, RV/TLC, and %DLCO) did not differ between the two groups, oscillometry parameters were significantly associated with the radiological presence of SAD [21]. The absence of correlations between oscillometric parameters and DLCO% or DLCO/VA in our pilot study likely suggests that oscillometry, focusing on small airway pathology, evaluates different pathophysiological aspects than diffusing capacity in this population of patients with mild ILD and may be complementary in the holistic evaluation of this population.

Our study showed that in patients without radiological evidence of obstruction, R5-19 captured SAD abnormality in one-third, correlated with disease severity and the prognostic score CPI. SAD may play a role in the worsening of respiratory symptoms and contribute to mortality risk in more advanced ILD disease, but importantly, early involvement of small airways in the disease course may imply its potential role in pathogenesis of pulmonary fibrosis [4,5]. Despite the small number of participants and the cross-sectional design of this study, our data are derived from a well-characterized population of patients with early pulmonary fibrosis. We excluded patients with pulmonary fibrosis in the context of collagen vascular disease or hypersensitivity pneumonitis, where SAD may be a cardinal pathological and radiological feature caused by different mechanisms from idiopathic fibrotic diseases. In contrast to IPF, small airway dysfunction is well recognized in rheumatoid arthritis and Sjogren’s syndrome, where obliterative and follicular bronchiolitis are common findings; similarly, in hypersensitivity pneumonitis (HP), granuloma formation and lymphocytic infiltrates lead to small airway obstruction [22]. Our results show that small airways may be abnormal in early pulmonary fibrosis as assessed by R5-19, irrespective of smoking habit, and this finding correlates with FVC and the CPI. Further research focused on small airways, and their epithelium is likely warranted in the context of pulmonary fibrosis, which may stimulate exploration of new treatable traits. Small airway-targeted treatment via aerosolized drug delivery onto the airway epithelium could be a therapeutic option in the early stage of pulmonary fibrosis [23].

The small sample size is a potential limitation of our pilot study, yet the identification of patients with mild ILD and the meticulous evaluation of these patients, including pulmonary function testing and forced oscillometry on the same day at the time of the initial diagnosis supports the validity of our observations. The cross-sectional design of this study represents an additional limitation, and future prospective cohort studies in larger populations are certainly needed to identify the role of oscillometry in the evaluation and management of patients with ILD.

In conclusion, this is the first physiological study to our knowledge, investigating patients with early pulmonary fibrosis, with no evidence of comorbid obstructive disease, focusing on small airways. Our data highlight that oscillometry is an easy-to-use technique revealing early mechanical alterations caused by pulmonary fibrosis and captures SAD, a sensitive marker of peripheral lung pathology, which may have prognostic implications for ILD patients.

## Figures and Tables

**Table 1:** Correlations of oscillometry parameters with pulmonary function test parameters.

	R5 Act.		X5 Act.		Fres Act.		AX act.		R5-19 Act.	
Variables	r	*p*	r	*p*	r	*p*	r	*p*	r	*p*
FEV_1_, % pred.	−0.290	0.096	0.374	0.064	−0.396	**0.020**	−0.364	**0.034**	−0.240	0.172
FEV_1_, L	−0.601	**<0.001**	0.666	0.224	−0.552	**0.001**	−0.500	**0.003**	−0.483	**0.004**
FVC, % pred.	−0.287	0.099	0.397	**0.047**	−0.454	**0.007**	−0.515	**0.002**	−0.254	0.148
FVC, L	−0.588	**<0.001**	0.671	**<0.001**	−0.562	**0.001**	−0.515	**0.002**	−0.481	**0.004**
FEV_1_/FVC, %	0.218	0.216	−0.289	0.549	0.243	0.166	0.245	0.163	0.174	0.324
FEF25-75, % pred.	−0.366	**0.033**	0.337	0.736	−0.228	0.195	−0.166	0.347	−0.370	**0.021**
DLCO, % pred.	−0.134	0.458	0.208	0.405	−0.251	0.159	−0.184	0.304	−0.234	0.190
DLCO/VA, % pred.	0.084	0.680	0.069	0.767	0.049	0.785	0.081	0.654	−0.085	0.639
RV/TLC, % pred.	0.074	0.680	−0.106	0.750	0.036	0.842	0.081	0.653	−0.017	0.924
RV/TLC, %	0.076	0.690	−0.145	0.973	0.238	0.205	0.159	0.401	0.131	0.468
TLC, % pred.	−0.324	0.066	0.448	0.100	−0.479	**0.005**	−0.405	**0.019**	−0.311	0.078

Bold numbers indicate statistically significant values.

## Data Availability

The data are available from the authors upon request.
